# Supplementary material for: c-Abl Tyrosine Kinase Is Regulated Downstream of the Cytoskeletal Protein Synemin in Head and Neck Squamous Cell Carcinoma Radioresistance and DNA Repair
Source: Int J Mol Sci. 2020 Oct 1;21(19):7277. doi: 10.3390/ijms21197277 (PMC7583921; doi:10.3390/ijms21197277)
Supplement: Supplementary file 1 [file ijms-21-07277-s001.pdf]

International Journal of
Molecular Sciences

c-Abl tyrosine kinase is regulated downstream of the cytoskeletal protein synemin in head and neck squamous cell carcinoma radioresistance and DNA repair

Sara Sofia Deville, Luis Fernando Delgadillo and Anne Vehlow and Nils Cordes

Supplementary Figures

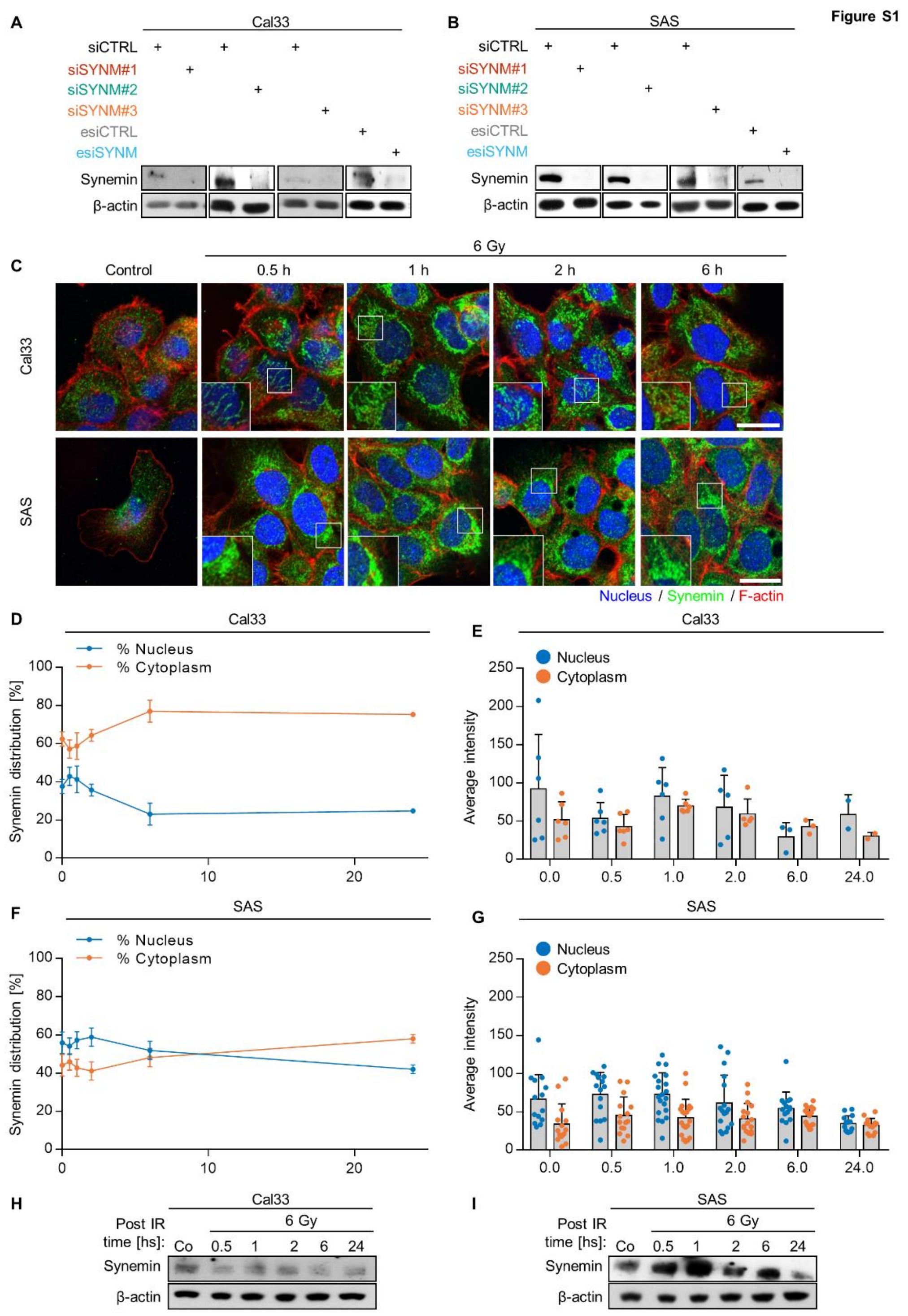

Figure S1. Characterization of synemin upon X-Ray irradiation. A, B, Knockdown efficiencies using three different siRNAs and one esiRNA for synemin silencing in Cal33 (**A**) and SAS (**B**) cells. **C,** Immunofluorescence staining of synemin (green) and its kinetics in Cal33 and SAS cells upon 6 Gy X-rays. Cells were counterstained for F-actin (red) and nucleus (blue) (bar, 20 μm). **D-G,** Kinetic analyses using Fiji software of confocal images showing synemin´s intracellular distribution in Cal33 and SAS cell lines. Ratio of mean fluorescence intensity of nuclear to cytoplasmic localization (**D,F**) and average intensity (**E,G**) were determined using the Intensity Ratio Nuclei Cytoplasm Tool plugin (NIH, USA). **H,I,** Western blots of synemin expression kinetics in whole cell lysates from Cal33 and SAS cells post 6 Gy irradiation. β-actin served as loading control.

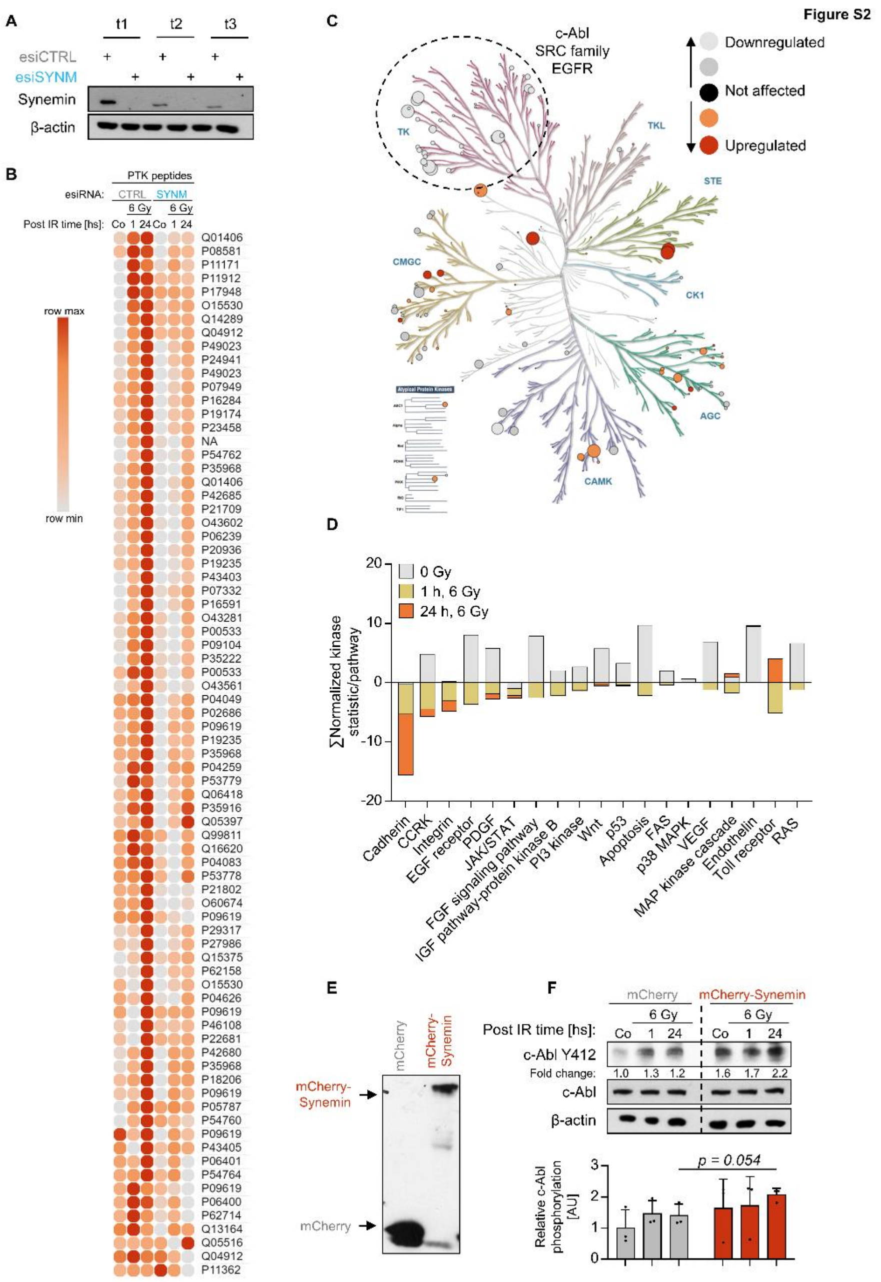

Figure S2. Synemin regulates tyrosine kinase activity, in particular c-Abl kinase. A, Western blots of knockdown efficiencies of samples employed for PAMgene-based kinome analysis. β-actin served as loading control. **B,** Heatmap of phosphorylated peptides from tyrosine kinases of control and synemin knockdown samples before and after 1 and 24 h X-ray irradiation (n=3). **C,** Kinase family tree of down- and up-regulated kinases 24 h post irradiation and synemin knockdown. Black dotted circle shows tyrosine kinases. **D,** Pathway classification of the de-regulated kinases using Panther analysis (http://www.pantherdb.org/). The mean kinase statistic of each kinase involved in the same pathway was summed to obtain an overall pathway related kinase statistic. **E,** Western blot on whole cell lysates from mCherry-Synemin transfectants at different time points post 6 Gy X-ray irradiation. β-actin served as loading control. **F**, Western blot of c-Abl expression and phosphorylation from whole cell lysates of synemin-overexpressing cells. Densitometries of Western blots shown in "E" from synemin-depleted, 6-Gy irradiated cells showing phosphorylated forms of c-Abl (n=3). Phosphorylation levels were calculated relative to the total amount of c-Abl. Data are presented as mean ± SD (n = 3; two-sided t-test).

Figure S3

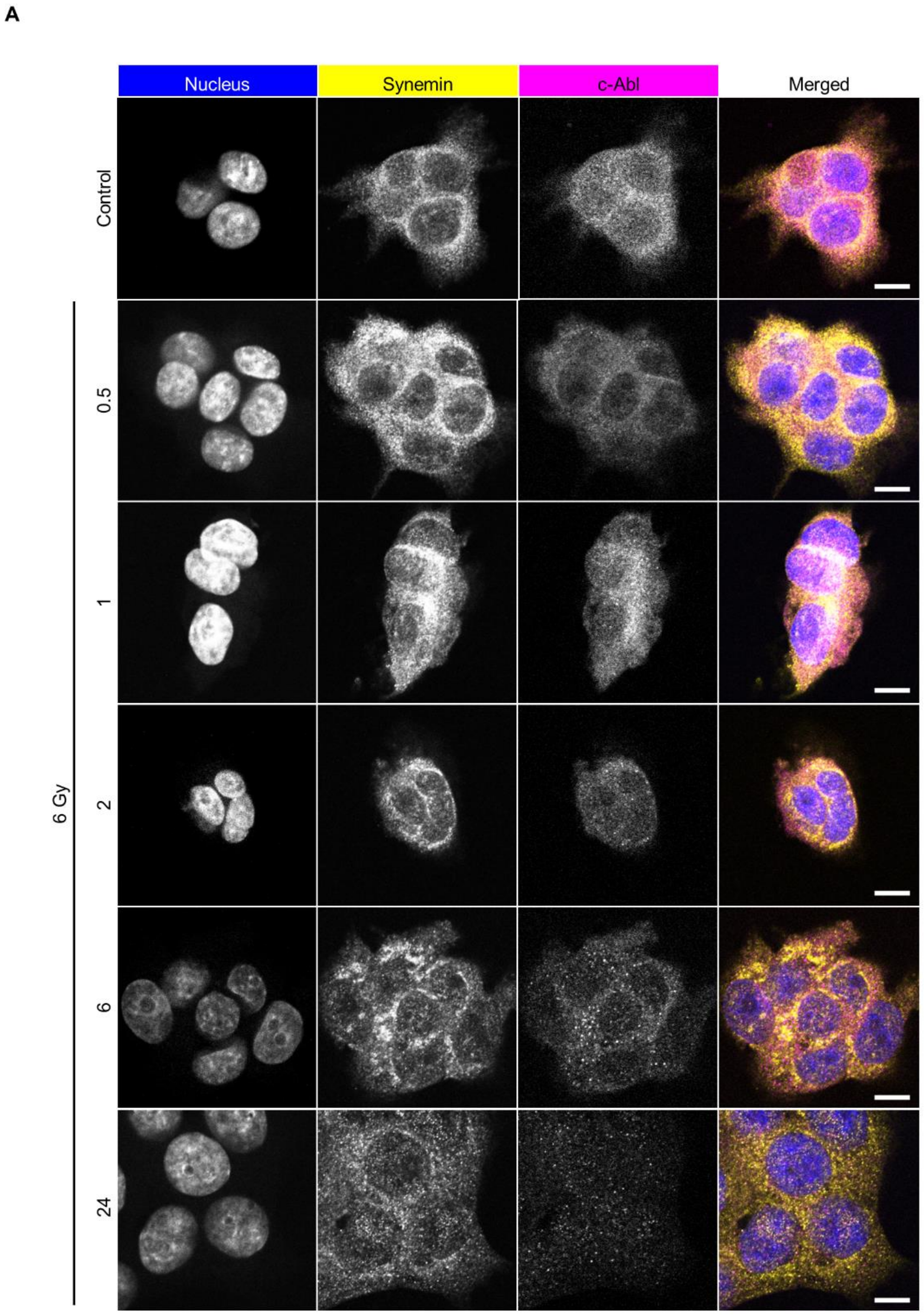

Figure S3. Synemin/c-Abl colocalization after X-ray exposure. **A,** Immunofluorescence staining of synemin and c-Abl in SAS cells after 6 Gy irradiation. Samples were fixed and analyzed at different time points (0.5, 1, 2, 6 and 24 h) post X-ray exposure (bar, 20 μm).

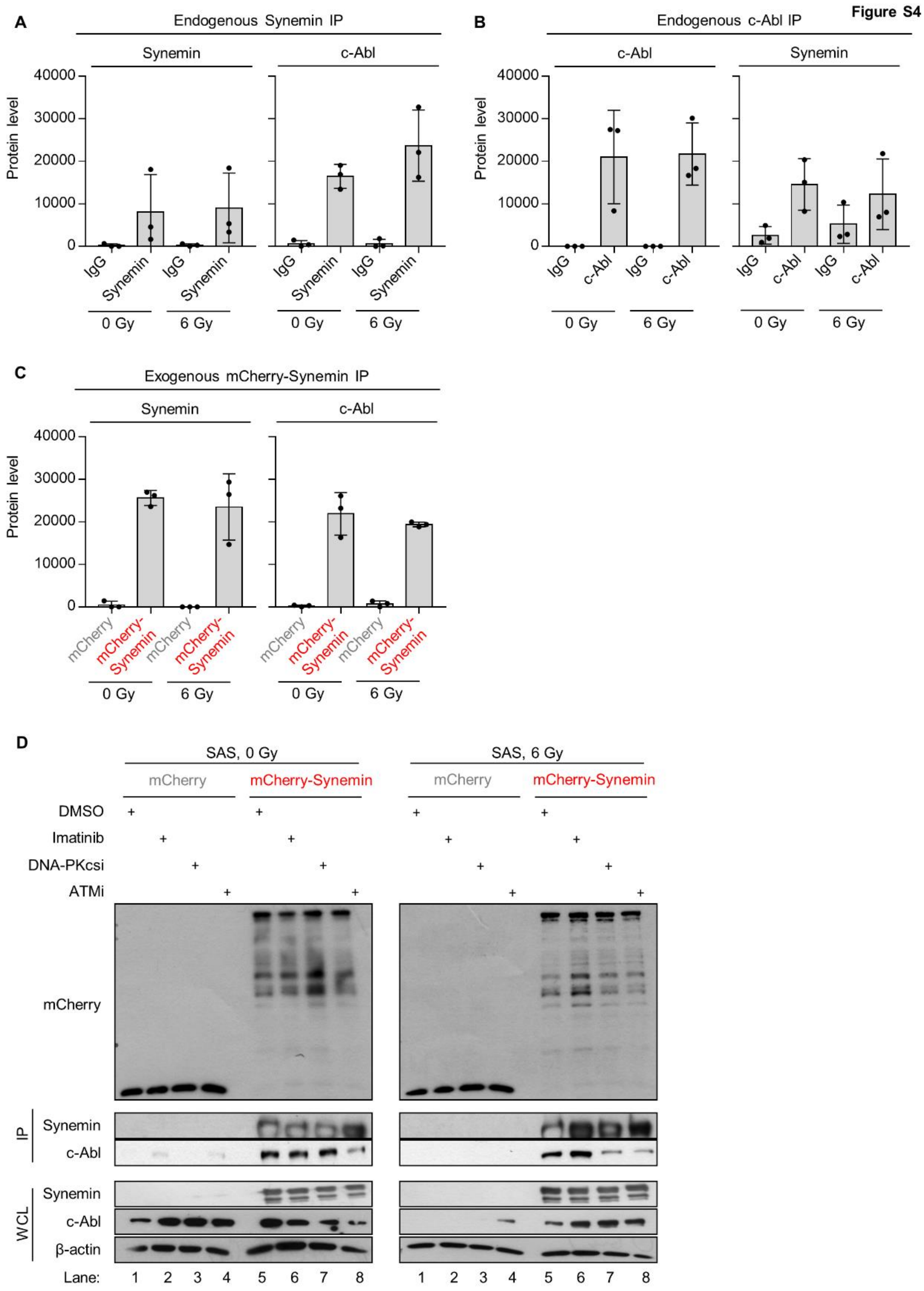

Figure S4. Synemin/c-Abl interact in an ATM-dependent manner. **A**, Densitometries of endogenous synemin immunoprecipitations (IPs) from Figure 4B. **B**, Densitometries of endogenous c-Abl IPs from Figure 4C. **C**, Densitometries of exogenous mCherry (empty vector) and mCherry-Synemin IPs from Figure 4D. **D**, Western blots on mCherry immunoprecipitates from mCherry-SAS and mCherry-Synemin-SAS cells after a 1-h pretreatment with Imatinib, DNA-PKcsi or ATMi alone or in combination with 6 Gy X-rays. IP, immunoprecipitate; WCL, whole cell lysates.
